# Axonal transport of botulinum toxin A from periphery to CNS in sensory and motor nerves

**DOI:** 10.1186/2050-6511-13-S1-A84

**Published:** 2012-09-17

**Authors:** Ivica Matak, Lidija Bach-Rojecky, Boris Filipović, Peter Riederer, Zdravko Lacković

**Affiliations:** 1 Laboratory of Molecular Neuropharmacology, Department of Pharmacology, University of Zagreb School of Medicine, 10000 Zagreb, Croatia; 2 Department of Pharmacology, University of Zagreb School of Pharmacy, 10000 Zagreb, Croatia; 3 Department of Psychiatry, Psychosomatics and Psychotherapy, University of Würzburg, 97070 Würzburg, Germany

## Background

Botulinum toxin A (BTX-A) has been approved for treatment of movement disorders and migraine. The widely assumed peripheral mechanism of action has been questioned by recent studies which demonstrated an axonal transport in the facial nerve and within central nerves. Findings in our laboratory suggested a central antinociceptive activity following axonal transport in the sciatic nerve.

## Methods

To characterize the axonal transport of BTX-A, the toxin's enzymatic activity in the CNS was assessed using immunofluorescent detection of its cleaved substrate synaptosomal-associated protein 25 (SNAP-25) following injections into the rat whisker pad and hind-paw, intramuscular injection into the gastrocnemius and intraneural injections into the sciatic nerve. Intraneural and intraganglionic colchicine was employed to block axonal transport. To investigate the significance of axonal transport for antinociceptive activity of BTX-A, we assessed the effect of peripheral and intraganglionic injections of low dose BTX-A in orofacial formalin-induced pain in rats.

## Results

Following whisker pad BTX-A injection, cleaved SNAP-25 was observed in the dorsal horn of the medulla. Cleaved SNAP-25 following subcutaneous, intramuscular and intraneural toxin injection in rat hind limbs was observed in corresponding segments of ipsilateral dorsal and ventral horn. Central SNAP-25 cleavage following BTX-A injection into the sciatic nerve was prevented by colchicine. In the ventral horn, BTX-A protease was localized within cholinergic neurons. Facial and intraganglionic injections of BTX-A prevented orofacial pain dependent on axonal transport in the trigeminal sensory nerve.

## Conclusions

Our results suggest that the axonal transport of BTX-A in different sensory and motor nerves commonly occurs after peripheral administration. Axonal transport in sensory neurons followed by central enzymatic activity is involved in botulinum toxin's antinociceptive effects. The possible functional role of axonal transport in motor neurons remains to be examined.

